# Rapid Catalytic Reduction of 4-Nitrophenol and Clock Reaction of Methylene Blue using Copper Nanowires

**DOI:** 10.3390/nano9070936

**Published:** 2019-06-28

**Authors:** Aina Shasha Hashimi, Muhammad Amirul Nazhif Mohd Nohan, Siew Xian Chin, Sarani Zakaria, Chin Hua Chia

**Affiliations:** 1 Materials Science Program, Faculty of Science and Technology, Universiti Kebangsaan Malaysia, 43600 Bangi, Selangor, Malaysia; 2 ASASIpintar Program, Pusat GENIUS@Pintar Negara, Universiti Kebangsaan Malaysia, 43600 Bangi, Selangor, Malaysia

**Keywords:** catalytic activity, clock cycle, acetic acid treatment, metal nanowires, *p*-nitrophenol, reduction of nitro compounds

## Abstract

Copper nanowires (CuNWs) with a high aspect ratio of ~2600 have been successfully synthesized by using a facile hydrothermal method. The reductions of 4-nitrophenol (4-NP) to 4-aminophenol (4-AP) and methylene blue (MB) to leucomethylene blue (LMB) by using sodium borohydride (NaBH_4_) were used as models to test the catalytic activity of CuNWs. We showed that by increasing the CuNWs content, the rate of reduction increased as well. The CuNWs showed an excellent catalytic performance where 99% reduction of 4-NP to 4-AP occurred in just 60 s by using only 0.1 pg of CuNWs after treatment with glacial acetic acid (GAA). The rate constant (k_app_) and activity factor (K) of this study is 18 and ~10^10^ fold in comparison to previous study done with no GAA treatment applied, respectively. The CuNWs showed an outstanding catalytic activity for at least ten consecutive reusability tests with a consistent result in 4-NP reduction. In clock reaction of MB, approximately 99% of reduction of MB into LMB was achieved in ~5 s by using 2 μg CuNWs. Moreover, the addition of NaOH can improve the rate and degree of recolorization of LMB to MB.

## 1. Introduction

Metal nanocrystals have attracted the attention of many researchers for the applications such as catalysts, sensors, antibacterial, and flexible and transparent electrodes [1,2,3,4,5,6,7,8]. Metal based nanoparticles have unique properties such as high numbers of vacant sites and high surface area which is why they are widely used for the removal of variety of toxic substances [9]. There are several parameters that can affect the catalytic activity of metal nanocrystal such as shape, size, and degree of dispersion. Metal particles at nanoscale often have significant catalytic properties compared to their bulk counterparts thanks to their increased surface-to-volume ratio [10]. One-dimensional (1D) nanocopper has received great attention due to its high abundance and low cost [11].

A recent trend in nanocrystals applications is using them as nanocatalysts to treat toxic substances such as nitroaromatic compounds. 4-nitrophenol (4-NP) is an example of toxic substance that exists in industrial and agricultural wastewaters, and has been classified as a priority toxic pollutant by the U.S. Environmental Protection Agency (EPA). [12] There are many methods which have been employed to reduce 4-NP such as photocatalytic [13], chemical oxidation [14], and the Fenton oxidation reaction [15]. Due to its simplicity, one of the most popular methods is by NaBH_4_ to reduce 4-NP to 4-AP. The reduction of 4-NP (E^0^_(4-NP/4-AP)_ = −0.76 V) by NaBH_4_ (E^0^_(H3BO3/BH4_^−^_)_ =−1.33 V) is thermodynamically favorable but there is a high kinetic barrier between the mutually repelling negative ions 4-NP and BH_4_^−^ in the absence of an effective catalyst [16]. The reduction of 4-NP by NaBH_4_ has become a widely used model reaction to assess catalytic properties of nanostructure materials.

Metals such as Au [3], Ag [13], Cu [17], and Pt [14] have been used in different shapes and sizes to reduce 4-NP. There are also studies that uses semiconductor catalysts such as ZnO [18] and CuO/TiO_2_ [19] to reduce 4-NP. There have been several studies of using Cu nanocrystal and bimetallic Cu nanostructures [20,21] as catalysts for the reduction of 4-NP. For example, Deka et al. successfully synthesized Cu nanoparticles (CuNPs) that can reduce 4-NP to 4-AP in 50 min [17]. Zhang et al. synthesized copper nanocrystals with different diameters and tested their catalytic performance in reduction of 4-NP [22]. They concluded that Cu nanocubes exhibit higher catalytic activity than Cu polyhedrons, illustrating that different morphologies of nanostructures may perform differently from one another. 

Oscillation between one or more components that occur in periodic motion is a complex reaction is called a chemical clock or oscillating reaction [23,24]. A clock reaction is usually based on a redox system and provides a visually reversible color change. The mechanism and features of a clock reaction may differ with different varieties of substrate. Some common examples of such reactions include the chlorate-iodine clock reaction [25], the molybdenum blue clock reaction [26], and the methylene blue (MB) clock reaction [27]. MB (tetramethylthionine chloride, C_16_H_18_ClN_3_S) is a blue colored thiazine dye that is water soluble. MB is blue when it is in an oxidizing environment and colorless as leucomethylene blue (LMB) when exposed to a reducing agent [28]. These redox properties of MB has several uses: As an analytical indicator [29], in rewritable paper [30], and as an oxygen indicator to ensure quality control in food industries [31,32]. This reaction also used to observe the catalytic performance of nanomaterials [33,34].

Clock cycles of MB has been demonstrated with several different types of catalysts and methods. Jiang et al. showed that ultrathin Cu_7_S_4_ nanosheets effected the clock cycle reaction of MB with hydrazine [33]. Clock reaction of MB was also studied by Pal et al. by using Au-CuO composite as a catalyst and ascorbic acid as a reducing agent [34]. They showed that the reduction of MB can be altered by using different amounts of catalyst. When more catalyst is added, the rate of decolorization becomes faster, following the pseudo-first order kinetics. The colorless solution of LMB can regain its blue color by gentle shaking. This is due to the aerobic oxidation of LMB to MB.

However, there are several obstacles to supported metal catalysis such as agglomeration of nanocatalysts during catalyst recovery, and reduction of specific area after being embedded on the support. Furthermore, regeneration of metals and leaching of catalyst during catalyst recovery are also matters to take concern in [35]. In this paper, we synthesized CuNWs by a simple hydrothermal route and studied its catalytic performance in the reduction of 4-NP to 4-AP and clock reaction of MB to LMB. In addition, a facile treatment using GAA was conducted on CuNWs immobilized onto cotton cloth to improve the reusability and recoverability of the CuNWs in the catalytic reduction of 4-NP.

## 2. Materials and Methods 

### 2.1. Materials

Copper chloride dihydrate (CuCl_2_·2H_2_O, ≥99.0%), octadecylamine (ODA, C_18_H_39_N, ≥85.0%), sodium borohydride (NaBH_4,_ ≥98%), and chloroform (CHCl_3_, ≥99.8%) were obtained from Merck, Massachusetts, USA. Glucose (C_6_H_12_O_6_, ≥99.5%), methylene blue (MB, C_16_H_18_ClN_3_S, ≥95%), and ascorbic acid (AA, C_6_H_8_O_6_, ≥99%) were obtained from Sigma, St. Louis, MO, USA. Glacial acetic acid (GAA, CH_3_COOH, 99.85%) was obtained from HmbG Chemicals, Hamburg, Germany. 4-Nitrophenol (4-NP, C_6_H_5_NO_3_, 99%) was obtained from Acros Organics, Geel, Belgium. Sodium hydroxide (NaOH, 99%) was obtained from SYSTERM, Essex, UK. All cotton cloths (CC, 95% cotton) were cut to 0.5 × 1 cm^2^ for each reaction. All chemicals were used as received. All solutions were prepared with deionized water.

### 2.2. Synthesis of CuNWs

CuNWs were synthesized using a previous reported method with slight modifications [2]. Firstly, 5.6 mM of CuCl_2_·2H_2_O, 26.3 mM of ODA and 2.8 mM of AA were dissolved in 30 mL deionized water and sonicated for 15 min. After that, the obtained mixture was transferred into a Teflon-lined autoclave and sealed for 20 h at 120 °C. After the synthesis was done, the reddish brown solution was washed with chloroform using the method outlined by Qian et al [36]. The fluffy reddish product that formed after the washing was stored in chloroform at 4 °C.

### 2.3. Preparations of CuNWs Strips

Different amounts of CuNWs (0.1, 0.5 and 1 pg) were drop casted on a piece of CC. Each strip was treated with 10% GAA (GAA: isopropyl alcohol = 1:9) for 10 min by dip coating.

### 2.4. Catalytic Reduction of 4-NP and Clock Reaction of MB Using CuNWs Strips

A 5 mL aqueous solution containing 1 mM 4-NP was prepared. 0.5 mL of 50 mM NaBH_4_ was added into the solution. CC containing CuNWs was added to this solution and stirred at 200 rpm using magnetic stirrer. To monitor the reduction of 4-NP, the absorbance values at 400 nm for 4-NP were recorded using a UV–vis spectrometer (Jenway, 7315). The reduction reactions of 4-NP were done to observe the effect of catalyst content, initial concentration of NaBH_4_, and initial concentration of 4-NP. The experimental conditions for the clock reaction of MB were the same as catalytic reduction of 4-NP with a slight difference in the mass of catalyst used. For clock reaction, only 2 μg of CuNWs was tested. To monitor the reduction of MB, the absorbance values at 665 nm for MB were recorded using a UV–Vis spectrometer.

The catalytic reduction efficiency of 4-NP of the as-synthesized sample was calculated as follows:

(1) $$\mathrm{Reduction}\;\mathrm{or}\;\mathrm{conversion}\;(\%)\;=\;\frac{C_{0}-C_{t}}{C_{0}}\;\times \;100\%$$

(2) $$\ln\left[C\right]/\left[C_{0}\right]{\;=\;-k}_{\mathrm{app}}t$$

where C and C_0_ are the concentration of 4-NP at time t and 0, respectively. The reaction rate constant (k_app_) was determined from the linear plot of ln (C/C_0_).

### 2.5. Retreatment of CuNWs Using GAA

After each cycle, CuNWs strip was dipped into 10% GAA for 10 min. The reactions continued under the same parameters.

### 2.6. Characterizations

The morphologies of CuNWs were analyzed by a scanning electron microscope (FESEM, MERLIN ZEISS, Oberkochen, Germany) and transmission electron microscopy (TEM, Talos 120C Thermo Fisher, Waltham, MA, USA). For TEM measurement, a small amount of the sample was dispersed in chloroform and a drop of the dispersion was spread on a carbon-coated copper grid. The reduction of 4-NP and MB were measured using UV–vis spectrophotometry (Jenway 7315, Staffordshire, UK) at wavelengths of 400 nm and 665 nm, respectively. The chemical composition and crystal structure of the samples were examined by X-ray diffraction (XRD, Bruker D8 Advance, Coventry, UK) under Cu Kα radiation. The current flow of the CuNWs was analyzed using Scanning Probe Microscope (SPM, NTEGRA NT-MDT, Moscow, Russia) and was operated by using the scanning spreading resistance microscopy (SSRM) mode. CuNWs were dispersed in chloroform and spin coated on a fluorine-doped tin oxide (FTO) glass slide for the SSRM measurements.

## 3. Results and Discussion

### 3.1. Characterization of CuNWs

**Figure 1:**
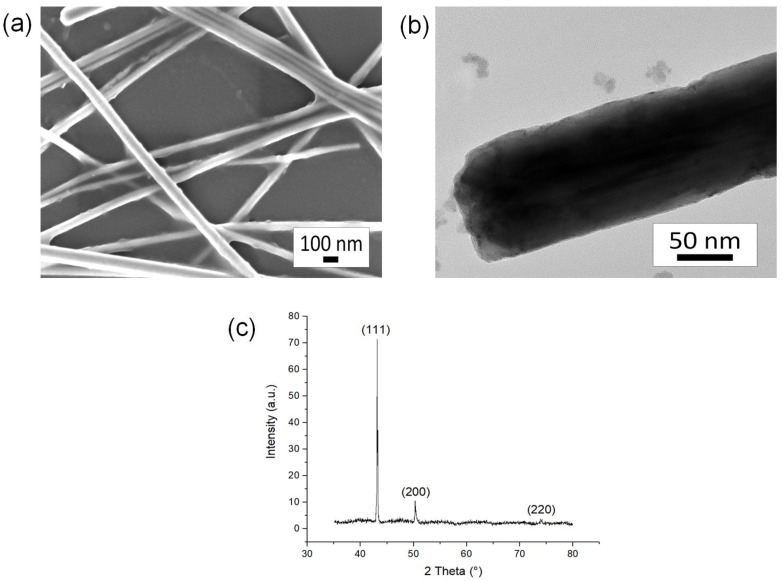
(**a**) FESEM image, (**b**) TEM image, and (**c**) XRD diffractogram of copper nanowires (CuNWs).

The morphologies and sizes of the CuNWs were investigated by FESEM and TEM. Figure 1a,b shows the overview image of the synthesized CuNWs by FESEM and TEM, respectively. The aspect ratio of the CuNWs obtained was ~2600. The growth mechanism of CuNWs was due to the Ostwald ripening mechanism which was described by Sun et al. [37,38]. At the beginning, the formation of two distinctive sizes of CuNPs might simultaneously occur in the reaction solution. Surface energies of the larger particles are less than the smaller ones as the reaction proceeds, which causes the small CuNPs to spontaneously dissolve into the solution and recrystallize on the larger ones [38]. Anisotropic growth is possible with the presence of capping agent which enables control of the morphology of NWs; capping agents act as the structure-directing agent by complexing the Cu(II) ion [39]. Figure 1c shows the XRD diffractogram of the CuNWs synthesized by hydrothermal reaction. The results showed three diffraction peaks of (111), (200), and (220) at 2θ = 43.2°, 50.3°, and 74.2°, respectively. All diffraction peaks are those of face-centered cubic Cu (JCPDS04-0836) [40].

### 3.2. Catalytic Reduction of 4-NP Using CuNWs

#### 3.2.1. Effect of CuNWs Content

**Figure 2:**
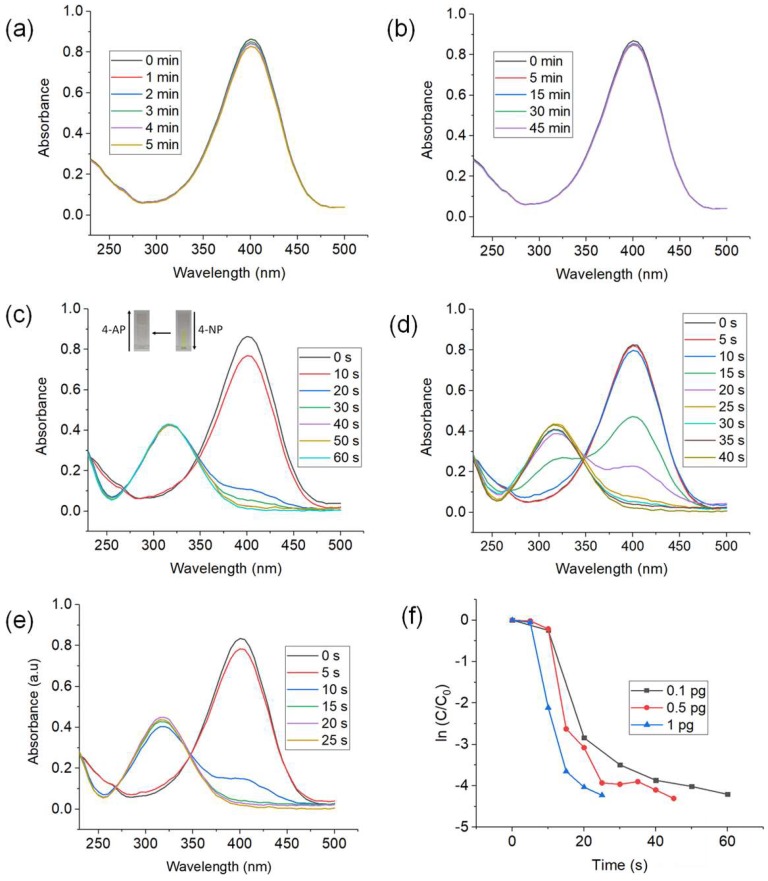
UV–vis spectra of 4-NP reduction reaction (**a**) no GAA treatment applied to CuNWs (**b**) no CuNWs added (**c**) 0.1 pg CuNWs (**d**) 0.5 pg CuNWs (**e**) 1 μg CuNWs added as catalyst, and (**f**) plot of ln (C/C_0_) against the reaction time for the reduction of 4-NP using different amount of CuNWs. (Reaction conditions: 5 mL of [4-NP] = 1 mM and 0.5 mL of [NaBH_4_] = 50 mM). The inset image in (**c**) is the color change of 4-NP to 4-AP.

To test the catalytic performance of the CuNWs, reduction of 4-NP to 4-AP was selected as the model reaction. The catalytic reaction does not occur when the CuNWs strips was not treated by GAA (Figure 2a). This might be due to the presence of a thin oxide layer and residual ODA presented on the surface of CuNWs [11] that prevent direct contact of reactants with the CuNWs. After CuNWs was treated with GAA for 10 mins, the reduction process occurred instantly. This shows that with GAA treatment, copper oxide, and residual capping agents can be removed from the surface of CuNWs without etching the underlying copper [2,41].

As can be seen from Figure 2b, the adsorption peak at 400 nm stayed almost unchanged in intensity for a long time which shows that the reduction of 4-nitrophenolate with only NaBH_4_ occurred very slowly. Amazingly, just by adding a minuscule amount of CuNWs (0.1 pg), the reduction process occurred instantaneously. As the peak of 4-nitrophenolate at 400 nm decreased, a new absorbance peak of 4-AP at 315 nm [20] emerged and increased with time. At the same time, the yellow color of 4-nitrophenolate changed to colorless, which also indicated the formation of 4-AP [42]. Figure 2c–e shows that by increasing the amount of CuNWs, the rate of catalytic reaction increases as well. Conversion of 4-NP to 4-AP up to 99% occurred in just 60, 40, and 25 s by using CuNWs loading of 0.1, 0.5, and 1 pg, respectively. 

Figure 2f shows the plot of ln (C/C_0_) against time for each of the CuNWs content tested. The concentration of NaBH_4_ can be considered as constant throughout the whole reaction due to its great excess in the reaction therefore, pseudo-first-order kinetics can be applied with respect to the concentration of 4-NP [17,43]. The rate constant for 0.1, 0.5, and 1 pg are 0.076, 0.11, and 0.2 s^−1^, respectively. It can be seen that as the mass of catalyst increases, the rate constant value increases as well. This shows that the efficiency of the reduction of 4-NP increased in correlation to the catalyst loading. The increase in total surface area and the number of reaction sites can be the cause of this enhanced catalytic activity [44,45]. Table 1 shows the list of rate constant of previously reported Cu-based catalysts for the reduction of 4-NP into 4-AP. The activity factor (K) was defined as the values of k_app_ obtained from the slope over the total weight of catalyst [21,46]. Both the k_app_ and K obtained from this study by using 0.1 pg CuNWs as catalyst shows a much higher value than the other form of Cu catalysts, indicating the excellent catalytic performance of CuNWs produced from this study.

In comparison to the catalytic reduction of 4-NP done by Sun et. al which also used CuNWs [21] as catalyst, both of the k_app_ and K values are lower than the ones obtained in this study. The extremely high k_app_ and K values could be related to the GAA treatment applied on CuNWs strip. The treatment applied could remove the oxide and residual capping layer, leaving the surface of CuNWs exposed to much easier and faster catalytic reactions. The capability of treatment using GAA was further proven by SSRM measurements by comparing the current signal of CuNWs before and after GAA treatment. Current flow of the CuNWs coated on FTO was increased from 3.45 nA to 7.88 nA after the GAA treatment. 

Electron transfer from the donor (BH_4_^−^) to acceptors (4-NP) can be accelerated by metal nanoparticles such as silver, gold and copper [14]. At room temperature, the liberation of hydrogen gas from NaBH_4_ by hydrolysis acts as a hydrogen source. The reaction can be catalytically enhanced by metals [14,48]. As stated before, without addition of catalyst, the reduction of 4NP occurred extremely slow and formation of 4-AP at absorbance peak 315 nm was not observed. The probable reaction steps for catalytic reduction of 4-NP to 4-AP are as follows:

Firstly, NaBH_4_ reduces water to hydrogen [49] which is as shown in Equation (3) below:

NaBH_4_ + H_2_O → NaBO_2_ + 4H_2_ (3)

The reduction process produces hydrogen gas which can be seen as bubbles in the solution. The isosbestic points can be found at 270 and 345 nm, and indicate that the sole product of the reaction is 4-AP [50,51]. The reduction of 4NP can be explained by using the Langmuir–Hinshelwood model of heterogeneous catalyzed reduction [49,52]. Borohydride ions are adsorbed and reacted with the surface of CuNWs to produce active hydrogen species. At the same, 4-NP in the solution diffuses onto the surface of the nanowires and reacts with the active hydrogen species to form 4-AP. By lowering the activation energy of the reaction, CuNWs are able to catalytically contribute to the reduction of 4-NP [53,54].

#### 3.2.2. Effect of 4-NP and NaBH_4_ Concentration

**Figure 3:**
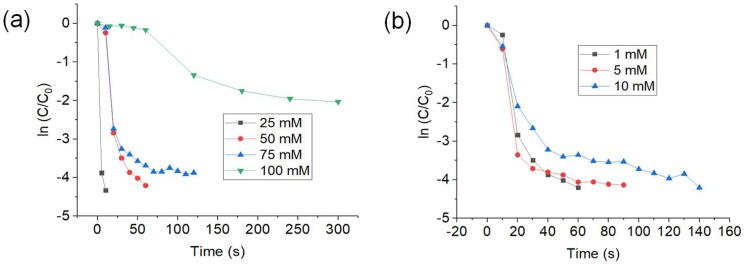
Plot of ln(C/C_0_) against the reaction time for the reduction of 4-NP using (**a**) different concentrations of NaBH_4_, and (**b**) different concentrations of 4-NP. Mass of CuNWs used was 0.1 pg.

Figure 3a shows the plot of ln (C/C_0_) against time for every concentration of NaBH_4_ tested. As the concentration of NaBH_4_ increases, the time taken for completion of reduction increases as well. The percentage of conversion also decreases with increase of NaBH_4_ used. For 25 and 50 mM of NaBH_4_, the time taken for 99% reduction is 10 and 60 s, respectively. Next, it takes 120 s for 75 mM of NaBH_4_ used to reach reduction percentage of 98%. Lastly, reduction percentage of 87% was reached in 300 s by using 100 mM NaBH_4_. The k_app_ also decreases with increasing concentrations of NaBH_4_ tested. The k_app_ for 25, 50, 75, and 100 mM are 0.433, 0.076, 0.027, and 0.0019 s^−1^, respectively. This is due to the inability of CuNWs to relay excess electrons [55,56].

Next, Figure 3b shows the plot of In (C/C_0_) against time for the concentrations of 4-NP tested. The time taken for 99% reduction of 4-NP is 60, 90, and 140 s for the concentrations of 4-NP 1, 5, and 10 mM, respectively. The k_app_ for the concentrations of 1, 5, and 10 mM are 0.076, 0.041, and 0.023 s^−1^, respectively. The decreasing trend in k_app_ could be due to the competition between the two reactants (4-NP and BH_4_^−^) to occupy the surface of CuNWs. Higher concentration of 4-NP has higher affinity to adsorb on the surface of CuNWs compared to BH_4_^–^ ion and this lessen the occupancy sites of BH_4_^–^ [57]. Liberation of hydrogen at the surface of CuNWs was limited due to the decreasing occupancy sites of BH_4_^–^ [58]. By using the Langmuir–Hinshelwood model, an efficient catalytic reduction through electron relay process is only achievable when both of the reactants are adsorbed onto the surface of catalyst in an almost equivalent amounts. Hence, the competition between 4-NP and BH_4_^–^ would be less which resulted in a faster reaction with higher rate constant values. Lower reduction rate at higher concentrations of 4-NP is observed due to the poor electron delay between the reactants [57].

#### 3.2.3. Recyclability Test and Retreatment of CuNWs with GAA

**Figure 4:**
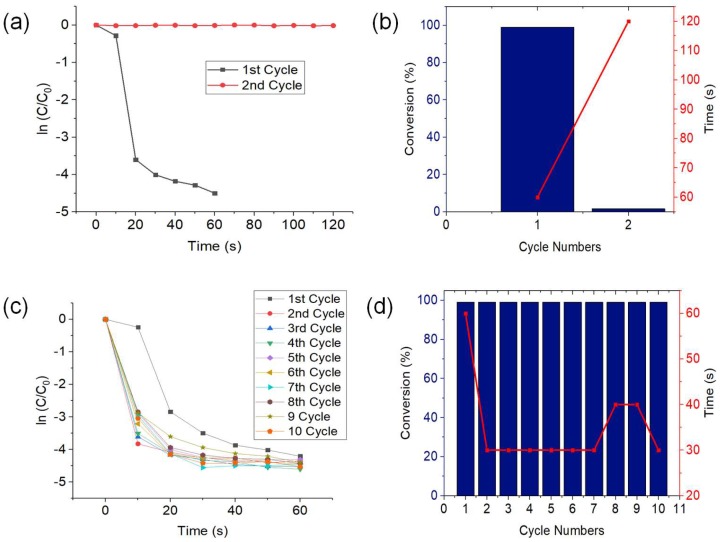
(**a**) Plot of ln(C/C_0_) against the time for the reduction (**b**) conversion percentage of 4-NP and reaction time without retreatment of GAA, and (**c**) plot of ln(C/C_0_) against the time for 10 cycles, and (**d**) conversion percentage of 4-NP and reaction time with retreatment of GAA.

For the practical application of catalyst, stability and recyclability is very important. To test the recyclability of CuNWs as catalyst, the same CuNWs strip was used to reduce several batches of 4-NP. Based on Figure 4a, it can be seen that the first cycle of conversion process progressed smoothly but there is almost no reduction of 4-NP in the second cycle. Conversion percentage of 4-NP in the second cycle was only 1.6% in 120 s (Figure 4b). This is probably due to the by-products blocking the active sites of CuNWs. Due to low conversion of the second cycle, retreatment of CuNWs using GAA was applied to see if there are any difference in the reduction progress. Figure 4c shows that the reduction of 4-NP can be continuously done by using the same CuNWs strip after retreatment with GAA before each cycle. This shows that the GAA treatment could remove all impurities from CuNWs surface which then enabled more catalytic reactions to occur.

Based on Figure 4d, the reduction process of the second cycle and above was faster (30 or 40 s) compared to the first cycle (60 s). This shows that when the first round was initiated, the assumed adsorption equilibrium between CuNWs and 4-NP was not yet totally established [59]. The recyclability results demonstrated that the catalytic performance of 0.1 pg CuNWs in a series of ten consecutive reduction reactions was stable and consistent without any significant difference. The reduction of 4-NP to 4-AP remained at 99% for the entire ten cycles. This shows that the small amount of CuNWs adhered extremely well to CC which resulted in good recoverability with high stability of catalytic activity. Videos S1 and S2 show that the catalytic reduction of 4-NP using CuNWs by using 0.1 pg of CuNWs and 25 mM of NaBH_4_. Video S1 shows that the rapid reduction of 4-NP using CuNWs as catalyst with a slight agitation by hand movement. Rapid continuous reduction of 4-NP can also be done as shown in Video S2.

### 3.3. Clock Reaction of MB

#### 3.3.1. Decolorization of MB using Sodium Borohydride (NaBH_4_) as Reducing Agent

To further test the catalytic activities of CuNWs, clock reaction of MB to LMB in the presence of NaBH_4_ was carried out. The blue color of solution is MB^+^ which is the water soluble oxidized form of MB [24]. The reduction of MB can be observed at the maximum absorption wavelength of 665 nm [60]. When there is no catalyst added to the reaction, there is a very small reduction in maximum intensity value which shows that the reduction of MB with only NaBH_4_ is extremely slow. The reduction of MB is kinetically restricted without catalyst, even though it is thermodynamically favorable. This is caused by the large potential difference between donor and acceptor molecules [61].

**Figure 5:**
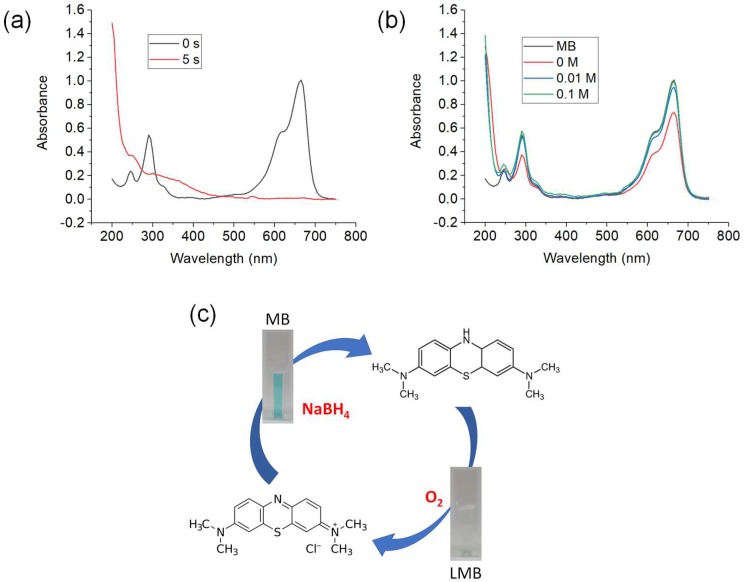
(**a**) UV–vis spectra of MB reduction reaction by NaBH_4_ using 2 μg of CuNWs as catalyst, (**b**) successive UV–vis spectra of recolorization of LMB using 2 μg of CuNWs by using different concentrations of NaOH (reaction conditions: 5 mL of [MB] = 5 mg/L and 0.5 mL of [NaBH_4_] = 50 mM), and (**c**) the schematic diagram of clock reaction of MB.

Like silver, copper can transfer electrons between donor and acceptor species efficiently as it is a good electrical conductor. The catalytic process can be explained in terms of a redox mechanism where the transfer of electrons from donor BH_4_^–^ ions to the acceptor (MB) is mediated by metal nanowires. This type of phenomenon where nanocrystal acts as redox catalyst is known as the “electron relay effect” [62]. Indication of reduction progress was evidenced by the color shift from blue to colorless. In this study, it was difficult to monitor the reduction of MB spectrophotometrically because of the promptness of color change in the presence of catalyst. Figure 5a shows the UV–vis spectra of decolorization of MB to LMB. The reduction percentage of MB to LMB in 5 s by using 2 μg CuNWs was 99%.

The reduction reaction of MB to LMB is a two-electron process. The electron delocalization length of MB is reduced, and p-conjugation breaks due to the reduction of the double bond in the heterocyclic ring. Next, two electrons are transferred to MB^+^ by NaBH_4_ [27]. By decreasing the concentration of dissolved oxygen in water, the amount of NaBH_4_ facilitates the formation of LMB [63]. To confirm the effect of catalyst on the oxidation of LMB to MB, CuNWs were removed immediately after the solution had turned colorless. The color changes back to blue which shows that the backward oxidation process could occur even after removal of catalyst. This verifies that the catalyst did not take part in the oxidation of LMB to MB [63].

The recolorization of MB by aerial oxidation doesn’t fully oxidize LMB to MB. This is probably due to the small amount of oxygen presence in dry air which is only about 21% [64]. Previously, Liu et al. reported that by the color of MB can return by using oxygen of 1 bar pressure [65], therefore in this study, NaOH is used instead of oxygen gas. After the complete conversion of MB to LMB, NaOH was added into the solution. Figure 5b shows the successive UV–vis spectra of different concentrations of NaOH added into LMB solution. Initially, 5 mg/L of MB has a maximum absorbance of 1.008 at λ_665_. After that, CuNWs strip was inserted into MB solution and complete decolorization process can be observed by the bleaching of blue color. In absence of NaOH, the highest absorbance value obtained after oxidation of LMB was 0.736 which shows that the MB color does not fully return.

It can be seen from Figure 5b that when NaOH is added into the solution, the maximum absorbance value at λ_665_ significantly increased. This shows that NaOH could help in the oxidation of LMB to MB. The absorbance value at λ_665_ rose to 0.948 and 0.9967 when 0.01 and 0.1 M of NaOH was added, respectively. Both are of higher absorbance value compared to the absence of NaOH. This also shows that higher concentration of NaOH could effectively oxidize more LMB to MB. This is probably due to the hydroxyl ions from NaOH. The hydroxyl ions would create an oxygen rich environment for LMB to convert back to MB. When higher concentration of NaOH is added, the amount of hydroxyl ions increased [66] which affects the oxidation of LMB. Furthermore, the recolorization rate increased when NaOH is added. In absence of NaOH, it takes around 10 min for maximum conversion of LMB to MB in ambient condition. Just by adding 0.01 M NaOH, the highest absorbance can be obtained in 30 s and this is similar when 0.1 M NaOH is added. This shows that NaOH also helps in speeding up the recolorization process of MB. Figure 5c shows the schematic diagram of color switching between MB (blue) and LMB (colorless).

#### 3.3.2. Comparison of Decolorization of MB using Different Reducing Agents

The catalytic performance of clock reaction of MB using CuNWs as catalyst was further studied by comparing different reducing agents of different strength to observe any difference in the clock reactions. The reducing agent tested were glucose and AA which are also greener and less toxic compared to NaBH_4_. Similarly, when NaBH_4_ is used as reducing agent, there is a very little decrease in the absorbance intensity value without the addition of catalyst. By using 2 μg of CuNWs as comparison, both glucose and AA could not reduce MB and initiate the clock reaction in a short period of time as NaBH_4_. Reduction of MB by using glucose and AA progressed steadily through 30 min but the reduction is not as drastic and significant as when using NaBH_4_. The reason why glucose and AA could not initiate a swift reduction and clock reaction of MB like NaBH_4_ is probably due to the lower reducing strength. Table 2 shows the decolorization percentage of MB using glucose, AA, and NaBH_4_.

## 4. Conclusions

In summary, CuNWs were synthesized using a simple hydrothermal method. They were used as catalyst for the reduction of 4-NP to 4-AP. The CuNWs exhibit superior catalytic activity: at just a minuscule amount of 0.1 pg, 99% reduction of 4-NP to 4-AP occurred in only 60 s. We also show how treatment of CuNWs using GAA helped in the reduction of 4-NP. CuNWs exhibit efficient and stable catalytic performance in up to ten consecutive cycles after retreatment with GAA. Furthermore, CuNWs showed rapid decolorization of MB to LMB in just 5 s. We also studied how the addition of NaOH can be used to improve the oxidation of LMB to MB. Lastly, the reduction of MB in the presence of CuNWs as catalyst could not be achieved when using reducing agents of lower strength than NaBH_4_.

## Figures and Tables

**Table 1:** Studies on the reduction of 4-NP using various catalysts.

Catalyst	k_app_ (s^−1^)	Activity Factor, K (s^−1^ mg^−1^)	Reference
CuNWs (0.1 pg)	0.076	7.6 × 10^8^	This study
Cu@MnO_2_	0.01142	0.571	[12]
CuNPs (12.5 mg)	0.0016	0.00013	[17]
CuNWs	0.0042	0.046	[21]
CuNW–Ag	0.0067	0.074	[21]
CuCubes (9.5 nm)	0.0101	0.105	[22]
CuNanoplate	0.0095	0.136	[46]
Porous Cu microsphere	0.0043	0.072	[47]

**Table 2:** Comparison of the decolorization percentage of MB by using glucose, AA, and NaBH_4_ as reducing agent.

Reducing Agent	Time of Reaction (s)	Percentage of Decolorization of MB (%)
Glucose	1800	35
AA	1800	43
NaBH_4_	5	99

## Supplementary Materials

The following are available online at https://www.mdpi.com/2079-4991/9/7/936/s1, Video S1: Reduction of 4-NP CuNWs, Video S2: Rapid continuous reduction of 4-NP by using CuNWs.
